# The MDRD equation underestimates the prevalence of CKD among blacks and overestimates the prevalence of CKD among whites compared to the CKD-EPI equation: a retrospective cohort study

**DOI:** 10.1186/1471-2369-13-4

**Published:** 2012-01-20

**Authors:** Pradeep Arora, Srini Rajagopalan, Nilang Patel, Neha Nainani, Rocco C Venuto, James W Lohr

**Affiliations:** 1 Department of Medicine, V.A. Medical Center 3495 Bailey Ave., Buffalo, New York, 14215, USA; 2 Department of Medicine, State University of New York at Buffalo, 462 Grider Street, Buffalo, New York, 14215, USA; 3 Med Data Analytics, Inc., 5500 Main St, Williamsville, NY 14221, USA

## Abstract

**Background:**

Black individuals are far more likely than white individuals to develop end stage renal disease (ESRD). However, earlier stages of chronic kidney disease (CKD) have been reported to be less prevalent among blacks. This disparity remains poorly understood. The objective of this study was to evaluate whether the lower prevalence of CKD among blacks in early stages of CKD might be due in part to an inability of the MDRD equation to accurately determine early stages of CKD in both the black and white population.

**Methods:**

We conducted a retrospective cohort study of 97, 451 patients seen in primary care clinic in Veterans Integrated Service Network 2 (VISN 2) over a 7 year period to determine the prevalence of CKD using both the Modification of Diet in Renal Disease (MDRD) Study equation and the more recently developed CKD Epidemiology Collaboration (CKD-EPI) equation. Demographic data, comorbid conditions, prescription of medications, and laboratory data were recorded. Logistic regression and quantile regression models were used to compare the prevalence of estimated glomerular filtration rate (eGFR) categories between black and white individuals.

**Results:**

The overall prevalence of CKD was lower when the CKD-EPI equation was used. Prevalence of CKD in whites was 53.2% by MDRD and 48.4% by CKD-EPI, versus 34.1% by MDRD and 34.5% by CKD-EPI in blacks. The cumulative logistic regression and quantile regression showed that when eGFR was calculated by the EPI method, blacks were as likely to present with an eGFR value less than 60 mL/min/1.73 m^2 ^as whites. Using the CKD-EPI equation, blacks were more likely than white individuals to have stage 3b, 4 and 5 CKD. Using the MDRD method, the prevalence in blacks was only higher than in whites for stage 4 and 5 CKD. Similar results were obtained when the analysis was confined to patients over 65 years of age.

**Conclusions:**

The MDRD equation overestimates the prevalence of CKD among whites and underestimates the prevalence of CKD in blacks compared to the CKD-EPI equation.

## Background

The incidence and prevalence of both CKD and ESRD in the United States continue to increase [1]. Age-adjusted ESRD rates are much higher for black individuals than white individuals (998 versus 273 per million) [2]. This disparity persists even after controlling for hypertension, diabetes, demographic characteristics, socioeconomic status and access to health care [3,4]. However studies have shown that the prevalence of early stages of CKD is lower in the black population. The Reasons for Geographic and Racial Differences in Stroke (REGARDS) study, a nationally representative sample of individuals 45 years and older revealed that estimated GFR < 60 ml/min/1.73 m^2 ^was present in 49.9% of white participants compared to 33.7% of blacks [3]. The National Health and Nutrition Examination Survey (NHANES) III showed similar results [5]. Thus the relationship of the racial prevalence of CKD to ESRD is complex, and not dependent solely on the prevalence of CKD.

These previous studies used a single serum creatinine measurement to determine the estimated GFR, the presence or absence of CKD, and its staging. The Kidney Disease Outcomes Quality Initiative (KDOQI) definition of CKD requires the determination of at least 2 serum creatinine measurements 3 months apart to document the presence of CKD [6]. The above studies also employed the MDRD equation for determining eGFR, which has been shown to underestimate GFR at higher values [7-9]. The CKD-EPI equation was developed as a more accurate determination of the GFR [10] and has been found to correlate better with long term risk of end-stage renal disease and mortality in a middle aged population [11]. We determined the prevalence of different stages of CKD using both the MDRD and CKD-EPI equations among the black versus white Veteran population in Veterans Integrated Service Network 2 (VISN 2), a large cohort consisting of all Veteran patients in central and western New York, and compared the use of two versus one serum creatinine in these equations. The objective of this study was to determine whether the lower prevalence of CKD among blacks in early stages of CKD might be due in part to an inability of the MDRD equation to accurately determine early stages of CKD in both the black and white population.

## Methods

This study was approved by the Buffalo VA Institutional Review Board. Data was obtained from the VISN 2 network (180,503 patients). All patients who were seen in primary care clinic in VISN2 from 4/1/2001 till 4/2008 were screened to estimate GFR by MDRD and CKD-EPI equation. We defined CKD as an eGFR < 60 ml/min/1.73 m^2 ^using the first recorded serum creatinine during this time period. Proteinuria was not considered in the definition. Demographic data obtained included age, gender, race, weight, height, body mass index (BMI), smoking history, and marital status. Race was determined by patient self report. The following co-morbid conditions were obtained from the clinical problem list by International Classification of Disease- 9^th ^Revision (ICD-9 codes): myocardial infarction (MI), coronary artery disease (CAD), congestive heart failure (CHF), peripheral vascular disease (PVD), chronic obstructive pulmonary disease (COPD), depression, cancer, diabetes, dyslipidemia, and hypertension (Table 1). Laboratory values for low density lipoprotein (LDL), triglyceride (TG) and high density lipoprotein (HDL) were obtained within 6 months of initial serum creatinine.

**Table 1:** Demographics of final sample

	Black (%)	White (%)	p value for difference
Total # of Patients with ≥ 2 Labs	8.38%	91.62%	
Gender			
Male	93.32%	95.21%	< .0001
Female	6.68%	4.79%	
Age (years)			
20-39	11.21%	6.58%	< .0001
40-59	58.43%	35.26%	
60-69	13.29%	20.82%	
≥ 70	17.07%	37.33%	
BMI			
< 25	24.20%	19.13%	< .0001
25-30	34.93%	38.28%	
30-40	35.07%	37.08%	
> 40	5.79%	5.51%	
Per Capita Income ($)			
< 20,000	47.34%	17.21%	< .0001
20,000 - 25,000	26.48%	38.75%	
25,000 - 30,000	11.75%	24.03%	
> 30,000	11.62%	18.01%	

### Definitions and equations

Age was re-calculated at each serum creatinine measurement as the difference in years between the date of serum creatinine measurement and the date of birth. Patients were stratified based on GFR estimated by MDRD and CKD-EPI formulae.

The re-expressed MDRD [8] formula used was: eGFR = 175 × (Scr)^-1.154 ^× age^-0.203 ^× 0.742 (if female) × 1.212 (if black), where Scr is serum creatinine in mg/dl and age is expressed in years.

The CKD-EPI [10] formula used was: eGFR = 141 × min(Scr/k, 1)^α ^× max(Scr/k, 1)^-1.209 ^× 0.993^Age ^× 1.018(if female) × 1.159 (if black), where Scr is serum creatinine in mg/dl, k is 0.7 for females and 0.9 for males, α is -0.329 for females and -0.411 for males, min indicates the minimum of Scr/k or 1, and max indicates the maximum of Scr/k or 1.

Prevalence was calculated as the ratio of patients with CKD to the total number of patients with at least two eGFR measurements in the duration of follow up. Patients with only one measurement were excluded. CKD was classified into stages based on the KDOQI guidelines [6] and National Institute for Health and Clinical Excellence (NICE) guidelines [12] as follows: stage 3a: GFR 45-59 ml/min per 1.73 m^2^, stage 3b: GFR 30-44 ml/min per 1.73 m^2^, stage 4: GFR 15-29 ml/min per 1.73 m^2^, and stage 5: GFR < 15 ml/min per 1.73 m^2^.

The first recorded creatinine value was used as the index creatinine. CKD stages were stratified based on the index creatinine when only one value of creatinine was used. The stratification into the stages based on the 2 values was done only if both the eGFRs were less than 60 ml/min/1.73 m2 (minimum time before 2^nd ^measurement was 3 months). If the subsequent eGFR was more than 60 ml/min per 1.73 m^2 ^the patient was not considered to have CKD.

### Statistical Analysis

Descriptive statistics were produced for the overall population and for the black and white groups separately. The descriptive statistics included patient demographics (age, gender, marital status, per capita income group), clinical variables (BMI, HDL-C, LDL-C, triglycerides) and comorbid conditions: MI, CAD, CHF, PVD, COPD, depression, cancer, diabetes, dyslipidemia, and hypertension). Proportions of patients with the above characteristics in different eGFR categories were compared using χ^2 ^test. Logistic regression was used to determine the effects of baseline characteristics on CKD condition as well as on classification in a particular eGFR category with ≥ 90 ml/min per 1.73 m^2 ^as the reference category. The two methods (EPI and MDRD) of calculation of eGFR were compared using the Cronbach's alpha measure.

Racial differences were explored in several other ways. First we ran individual logistic regressions of each eGFR category with the ≥ 60 mL/min/1.73 m^2 ^category as the reference level. We computed both unadjusted and adjusted odds ratios for blacks. Next we ran the cumulative logistic regressions comparing patients at a given level of eGFR with patients above that level. Again we computed both unadjusted and adjusted odds ratios for the African-American group. The adjusted model included age, gender, COPD, cerebrovascular event, depression, cancer, diabetes, dyslipidemia, hypertension, BMI group, presence of any vascular disease and proteinuria. As the distribution of patients, especially blacks, was not normal, and we were interested in the lower end of the distribution of eGFR, quantile regression models were built to examine the change in the race parameter over different percentiles. These models adjusted for the same variables used in the logistic regression models but also included a fourth-order polynomial of age. As there were significant age differences between whites and blacks, sensitivity analyses were done for patients above age 65 years. All the analyses were performed using SAS 9.2 (SAS Institute, Cary, NC). Statistical significance was set α = 0.05.

## Results

**Figure 1:**
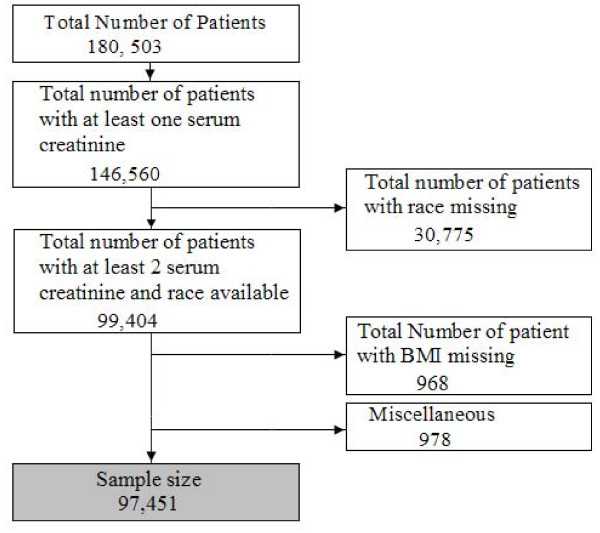
Study design.

**Figure 2:**
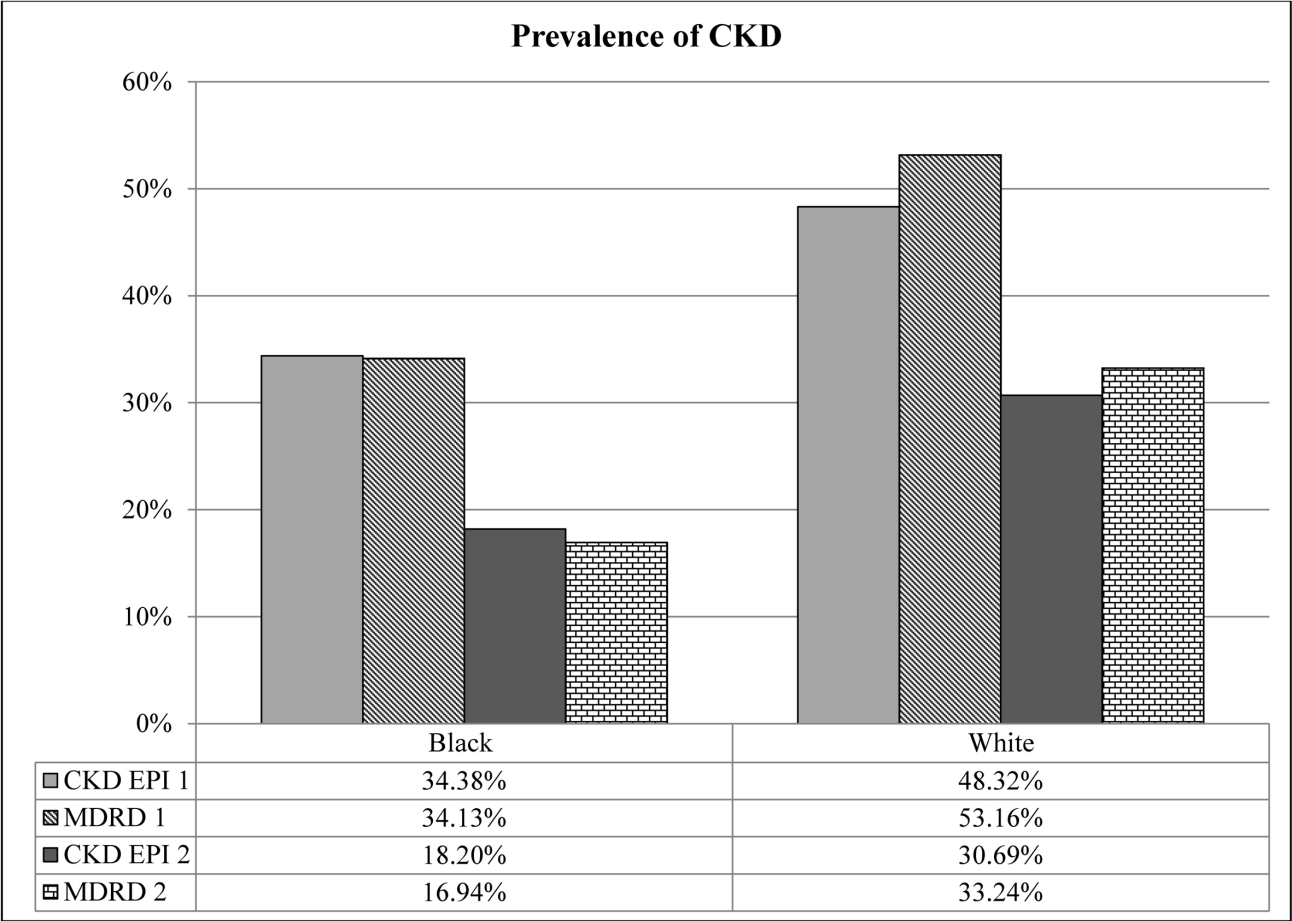
**Unadjusted prevalence of CKD in the study population when only 1 serum creatinine versus 2 serum creatinine measurements is used in the MDRD or CKD-EPI equation. The prevalence of CKD is reduced by 40% when using 2 serum creatinines**.

A total of 180,503 patients were screened from 4/1/01 to 4/1/08. The final sample size was 97,451 after excluding patients with only one serum creatinine measurement and those with race or date of birth missing (Figure 1). Demographics are shown in Table 1. The majority of patients were white and male. 28% of black patients were above 60 years of age compared to 58% of whites. Per capita income was lower for blacks. Table 2 shows the percentage of patients with CKD (eGFR < 60 ml/min per 1.73 m^2 ^by CKD-EPI) with various comorbidities. Looking at underlying cardiovascular disease, CAD, CHF, PVD, and CVA were all more likely to be present in white individuals with CKD. The prevalence of any diagnosis of vascular disease was 28.3% in whites and 15.3% in blacks. Even hypertension was more likely to be found in white individuals (62.2 vs. 59.8%). Diabetes and proteinuria were more commonly present in black individuals. Figure 2 shows the unadjusted prevalence of CKD in the study population when only 1 serum creatinine versus 2 serum creatinine measurements are used in the MDRD or CKD-EPI equation to define CKD. Overall the prevalence was reduced by almost 40% when 2 serum creatinine measurements are used compared to the single serum creatinine prevalence. We examined whether the likelihood of a patient having 1 versus 2 or more serum creatinine measurements performed differed by race and found no significant difference (data not shown).

**Table 2:** Patients with CKD (eGFR < 60 ml/min by CKD-EPI) with various comorbidities

	Overall (%)	Black (%)	White (%)	P value
Total # of Patients with ≥ 2 Labs		8.38%	91.62%	
MI	2.68%	1.15%	2.82%	< 0.001
CAD	17.42%	8.24%	18.26%	< 0.001
CHF	5.98%	4.29%	6.13%	< 0.001
PVD	9.55%	5.50%	9.92%	< 0.001
CVA	8.12%	6.49%	8.27%	< 0.001
Any Vascular Disease	27.25%	15.25%	28.34%	< 0.001
Depression	11.00%	14.09%	10.72%	< 0.001
Hypertension	62.05%	59.77%	62.26%	< 0.001
Dyslipidemia	59.44%	40.30%	61.19%	< 0.001
DM	27.76%	28.93%	27.66%	0.0137
Proteinuria	22.86%	29.42%	22.26%	< 0.001
HDL < 40 mg/dL	39.40%	29.31%	40.32%	< 0.001
LDL > 100 mg/dL	61.36%	59.73%	61.51%	0.908
TG > 200 mg/dL	21.83%	13.39%	22.60%	< 0.001

**Figure 3:**
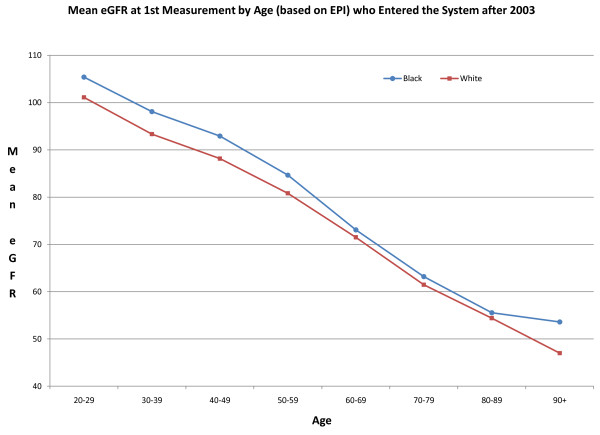
**Mean eGFR at first measurement after entry into VA VISN 2 health care system by CKD-EPI equation by race of patients**.

It has been suggested that one of the reasons a greater percentage of black patients are found to have ESRD as opposed to earlier stages of CKD is that blacks present to a primary care physician later in the course of their disease [13]. Figure 3 shows the mean eGFR at first serum creatinine determination by CKD-EPI equation by age of patients who entered the VA VISN 2 system after 2003. Although serum creatinine was higher among blacks, there was no difference in age-dependent eGFR between black and white patients with CKD at time of entry into the system.

The logistic regression for CKD showed that there was no difference between blacks and whites in terms of CKD prevalence (defined as less than 60 mL/min/1.73 m^2 ^as calculated by the CKD-EPI method) when adjusted for age and other comorbidities. This held true whether CKD was based on one or two measurements (data not shown). The adjusted cumulative logistic regressions showed that when eGFR was calculated by the CKD-EPI method, there was no difference (AOR 1.057, 95% CIs 0.981-1.139) between blacks and whites at a cutpoint of eGFR of 60 ml/min/1.73 m^2^, but that using the MDRD equation, there was a significantly lower risk (AOR 0.669, CIs 0.623 to 0.72) for blacks to have CKD at this cutpoint (Table 3). On the other hand, blacks were more than three times as likely as whites to have CKD stage 5 (eGFR < 15 mL/min/1.73 m^2^) (AOR 3.171 by CKD-EPI and 3.062 by MDRD) by both equations.

**Table 3:** Racial difference (Black vs. White) in eGFR distribution and odds ratio in cumulative logistic model

eGFR Category	CKD-EPI method	MDRD method
	Adjusted OR	Adjusted OR
≤ 89 Vs. ≥ 90	0.629 (0.595 to 0.665)	0.404 (0.382 to 0.427)
≤ 59 Vs. ≥ 60	1.057 (0.981 to 1.139)	0.669 (0.623 to 0.72)
≤ 44 Vs. ≥ 45	1.238 (1.114 to 1.376)	1.061 (0.951 to 1.184)
≤ 29 Vs. ≥ 30	1.616 (1.378 to 1.895)	1.498 (1.267 to 1.771)
< 15 Vs. ≥ 15	3.171 (2.458 to 4.09)	3.062 (2.35 to 3.989)

**Figure 4:**
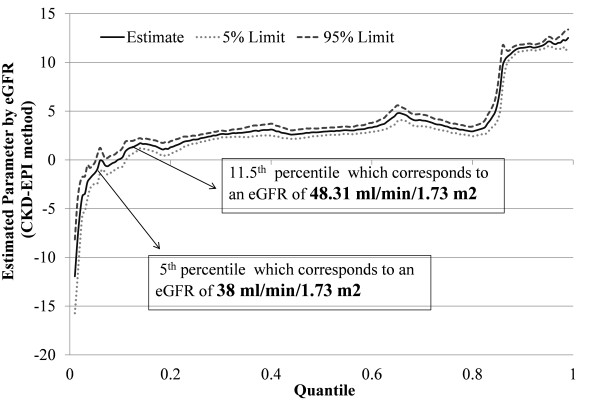
**Quantile regression adjusted for comorbidities and age (fourth order polynomial) for CKD-EPI equation**. The X axis displays eGFRs by quantiles (0-0.2 being the lowest 20% and 0.8- 1.0 being the highest 20%). The Y axis shows the difference in eGFR in ml/min in blacks compared to whites. For example, a black individual with an eGFR at the 40^th ^percentile would have an eGFR approximately 3 ml/min higher than a white individual by CKD-EPI method. Using the MDRD equation, a black at the 40^th ^percentile, would have an eGFR approximately 6 ml/min higher than a white individual.

Quantile regression adjusted for comorbidities and fourth order polynomial for age (Figure 4) showed that blacks generally had a significantly higher eGFR than whites by the CKD-EPI method except at the lower end of the eGFR distribution (below the 11.5^th ^percentile which corresponds to an eGFR of 48 ml/min/1.73 m^2 ^for the combined population) where there was no difference between blacks and whites. However, below the 5^th ^percentile, eGFR was significantly lower for black patients corresponding eGFR of 38 ml/min/1.73 m^2^). That is, blacks were likely to have lower eGFR using the CKD-EPI method in stages 3b, 4, and 5 CKD. On the other hand, in stage 3a or non-CKD conditions, whites were as likely as blacks to have a lower eGFR.

**Figure 5:**
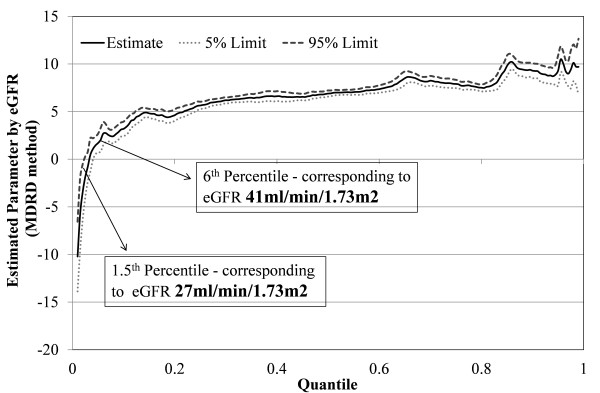
**Quantile regression adjusted for comorbidities and age (fourth order polynomial) for MDRD equation**. The X axis displays eGFRs by quantiles (0-0.2 being the lowest 20% and 0.8- 1.0 being the highest 20%). The Y axis shows the difference in eGFR in ml/min in blacks compared to whites. For example, a black individual with an eGFR at the 40^th ^percentile would have an eGFR approximately 3 ml/min higher than a white individual by CKD-EPI method. Using the MDRD equation, a black at the 40^th ^percentile, would have an eGFR approximately 6 ml/min higher than a white individual.

While the results for eGFR using the MDRD method (Figure 5) were similar to those for the CKD-EPI method, blacks had a higher eGFR value than whites above the 6^th ^percentile (corresponding to an eGFR of 41 ml/min/1.73 m^2^); and lower eGFR value below 1.5^th ^percentile (corresponding to an eGFR of 27 ml/min/1.73 m^2^). Therefore, in stage 3a or non-CKD conditions, whites were likely to have a lower eGFR. In stage 3b, both blacks and whites had a similar eGFR while blacks had a lower eGFR in stages 4 and 5. Similar results were obtained when the analysis was confined to patients above age 65.

**Figure 6:**
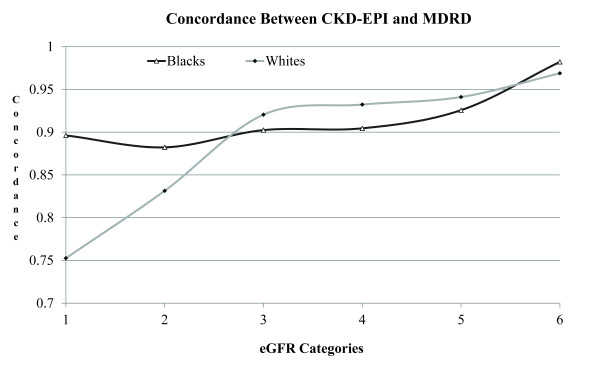
**Concordance between MDRD and CKD-EPI equations in black and white individuals according to eGFR categories**. On the X axis, in ml/min, categories 1-6 correspond to the following eGFRs (ml/min): 1 = > 90, 2 = 60-89, 3 = 45-59, 4 = 30-44, 5 = 15-29, and 6 = < 15.

To determine whether the CKD-EPI equation estimates GFR more accurately among blacks than the MDRD equation, we used Cronbach's alpha measure to compare these two methods of estimating GFR. Figure 6 shows the concordance between MDRD and CKD-EPI equations in black and white individuals according to eGFR categories. The concordance between MDRD and CKD-EPI equations for blacks was superior to that for whites. The excellent concordance between two equations in estimating GFR does not mean that these equations measure eGFR more accurately in blacks, only shows that they have a high degree of agreement in measurement.

## Discussion

We studied the prevalence of different stages of CKD among blacks and whites in > 180,000 patients who were seen in primary care clinic at VISN2, using MDRD and CKD-EPI equations. The cumulative logistic regression adjusted for age and other comorbidities showed that when eGFR was calculated by the CKD- EPI method, blacks were as likely as whites to present with an eGFR value less than 60 mL/min/1.73 m^2^. Using the CKD-EPI equation, blacks were more likely than white individuals to have stage 3b, 4 and 5 CKD. When eGFR was calculated by the MDRD method, the results were similar for values below 30 mL/min/1.73 m^2^. There was considerable difference between the two methods above this value. Similar results were also shown when quantile regression was used or analysis was confined to patients above age 65 years.

It is well established that the risk for ESRD is higher in black than white individuals, yet earlier stages of CKD have been found to be more prevalent in whites [1-5,14,15]. Clase et. al. examined the NHANES III database and found that the prevalence rate of CKD (eGFR < 60 ml/min/1.73 m^2 ^by the original MDRD equation [16]) in non-diabetic black males, black females, white males, and white females was 4.2%, 6.2%, 9.2%, and 17.8%, respectively [17]. Coresh et.al. evaluated the NHANES III database including diabetic individuals and found a prevalence of eGFR < 60 ml/min per 1.73 m^2 ^in 3.4% of black participants and 5.0% in white participants using a single measurement of serum creatinine in the simplified MDRD study equation [5]. In REGARDS, McClellan et. al. found that the prevalence of an eGFR < 60 ml/min per 1.73 m^2 ^was 33.7% in black patients and 49.9% in white patients using a single serum creatinine [3]. They examined the prevalence at different deciles of eGFR and using eGFR > 60 ml/min per 1.73 m^2^as the reference, found that the odds ratio for a low eGFR in blacks compared to whites increased as kidney function declined, with an odds ratio of .46 for eGFR 50 to 59 ml/min per 1.73 m^2 ^to an odds ratio of 2.56 for an eGFR of 10-20 ml/min per 1.73 m^2^. This relationship held true even after adjusting for age, gender, diabetes, hypertension, history of myocardial infarction or stroke, smoking status, and region of the country.

This inconsistency in prevalence between blacks and whites in early CKD versus ESRD remains unexplained. Several factors which have been proposed to explain this [3,4,14,18-29]. There may be more rapid progression of CKD in blacks due to less effective treatment of modifiable risk factors affecting the progression of CKD [18-22] or differences in genetic [23,24] and environmental [25] factors. Black patients with CKD may have a lower death rate and be more likely to reach ESRD. Newsome et al found that in a large cohort of CKD patients who had suffered a myocardial infarction, black patients had better survival after 3 years [26]. However, NHANES III data showed that black individuals with CKD under 65 years of age were more likely to die than white individuals, but there was no difference seen in individuals over 65 years of age [27]. Likewise, a study using the VA national database showed a higher mortality for black patients versus white patients at all levels of baseline GFR [14]. We did not examine mortality in this study.

Blacks may have higher prevalence of ESRD relative to CKD because they present to the health care system later in the course of kidney disease [13], we found no difference in baseline CKD-EPI eGFRs at time of entry to the VA system between black and white individuals (Figure 3). There may be differences in quality of care given to blacks compared to whites. A study showed decreased use of cardiovascular procedures in minorities which may affect morbidity and mortality from cardiovascular events [28]. However, a study of treatment regimens for CKD in the Department of Defense found similar compliance of care for stage 3 and 4 CKD in black and white individuals [29].

Another explanation for lower prevalence of CKD among the black population could be the lack of accurate tools to estimate GFR. The MDRD equation was derived from a large study of patients with chronic renal disease [16] which includes a correction factor of 20.5% for blacks for the same creatinine level compared to whites. This equation is widely used in clinical laboratories to estimate GFR. However it has been shown that this equation tends to underestimate GFR in healthy individuals [7,8]. The CKD-EPI equation was derived more recently in an attempt to rectify the fact that the MDRD equation underestimated measured GFR at higher values [10]. This equation was found to be more accurate than the MDRD equation, especially at higher GFRs. The sample populations used to develop the CKD-EPI equation and the MDRD equation had a limited number of elderly patients. However, the CKD-EPI population included 32% blacks compared to only 15% in the MDRD sample population. So it may be possible that CKD-EPI is a better equation for GFR estimation in blacks. Delanaye et. al. recently found a prevalence of stage 3 CKD of 11.04% using the MDRD equation versus 7.98% using the CKD-EPI equation in a screen of 1992 individuals [30]. Their study used a single creatinine measurement to define CKD and there were no black patients in their study population.

In the present study, when GFR was determined by CKD-EPI compared to MDRD, we found that the prevalence of earlier stages of CKD was not different in blacks compared to whites. Additional file 1 Table 4 shows why there were significant differences in classification of stages of CKD using the 2 formulas. The patients who were initially classified in different eGFR categories by CKD-EPI method were reclassified again by applying MDRD equation. In the overall patient group, 44.4% of patients who were classified into the > 90 ml/min per 1.73 m^2 ^eGFR group by CKD-EPI were re-classified by MDRD to the lower eGFR category of 60-89 ml/min per 1.73 m^2^. The overall difference in the prevalence of patients falling in to the 60-89 ml/min per 1.73 m^2 ^eGFR group increased by about 3.4% by using CKD-EPI (5.7% - 2.3% = 3.4%). The most noticeable finding was a large increase in the number of black individuals found to have stage 3a CKD (17% higher) when determined by CKD-EPIwho were classified to a no-CKD category (eGFR of 60-89 ml/min per 1.73 m^2^) by MDRD method. The number of white patients classified as stage 3a did not change. Similarly, 6% of the whites who were initially classified into an eGFR of 60-89 ml/min per 1.73 m^2 ^(No-CKD category) by CKD-EPI method were reclassified to an eGFR category of 45-59 ml/min per 1.73 m^2 ^(CKD stage 3a) by MDRD method. These observations suggest that the lower prevalence of CKD among black individuals is due to underestimation of earlier stages of CKD in blacks and overestimation of earlier stages of CKD among whites by MDRD method.

There are certain limitations to this study. First, we have not directly measured GFR. Proteinuria was not included in the evaluation, but this would be more critical to a study of progression rather than classification of CKD. Although the VHA is the largest integrated health care system in United States and utilizes a uniform data collection system, this is a retrospective study and some patients had to be excluded due to lack of information about gender and race. The study was done on individuals in the VA system, and therefore may not be applicable to the general population.

## Conclusions

This the first report of the racial prevalence of CKD in a large VA cohort using 2 serum creatinine measurements and employing the CKD-EPI equation to estimate GFR. Using an adjusted regression model, we found no difference in the prevalence of earlier stages of CKD in black individuals relative to white individuals. We found that the previously described higher prevalence of early stage CKD in whites may be accounted for by differences in classification of stages of CKD by the MDRD equation relative to the more recently derived CKD-EPI equation. The finding that the prevalence of early CKD is similar between the two races does not fully explain why ESRD is more prevalent in blacks. Further studies will be required to understand why this racial disparity persists.

## Competing interests

The authors declare that they have no competing interests.

## Authors' contributions

PA and JWL conceptualized the study and its objective. PA and JWL designed the study. PA, NN and NP extracted the data from VA database. SR analyzed the data statistically and contributed to the data's interpretation. PA wrote the manuscript; NP, NN, RCV, SR and JWL revised the manuscript critically and made substantial contributions to the content of the article. All authors read and approved the final manuscript.

## Pre-publication history

The pre-publication history for this paper can be accessed here:

http://www.biomedcentral.com/1471-2369/13/4/prepub

## Supplementary Material

Additional file 1 **Table 4: Reclassification of CKD-EPI group by MDRD equation**. Table 4 shows why there were significant differences in classification of stages of CKD using the 2 formulas. The patients who were initially classified in different eGFR categories by CKD-EPI method were reclassified again by applying MDRD equation. Click here for file

## References

[B1] Coresh J Selvin E Stevens LA Manzi J Kusek JW Eggers P Van Lente F Levey AS Prevalence of chronic kidney disease in the United States JAMA 2007 298 17 2038 2047 10.1001/jama.298.17.2038 17986697

[B2] United States Renal Data System USRDS 2009: Annual Report: Atlas of Chronic Kidney Disease and End Stage Renal Disease in the United States, Bethesda, MD National Institutes of Health. National Institute of Diabetes and Digestive and Kidney Diseases 2009

[B3] McClellan W Warnock DG McClure L Campbell RC Newsome BB Howard V Cushman M Howard G Racial differences in the prevalence of chronic kidney disease among participants in the Reasons for Geographic and Racial Differences in Stroke (REGARDS) Cohort Study J Am Soc Nephrol 2006 17 6 1710 1715 10.1681/ASN.2005111200 16641151

[B4] Xue JL Eggers PW Agodoa LY Foley RN Collins AJ Longitudinal study of racial and ethnic differences in developing end-stage renal disease among aged medicare beneficiaries J Am Soc Nephrol 2007 18 4 1299 1306 10.1681/ASN.2006050524 17329578

[B5] Coresh J Astor BC Greene T Eknoyan G Levey AS Prevalence of chronic kidney disease and decreased kidney function in the adult US population: Third National Health and Nutrition Examination Survey Am J Kidney Dis 2003 41 1 1 12 12500213 10.1053/ajkd.2003.50007

[B6] K/DOQI clinical practice guidelines for chronic kidney disease: evaluation, classification, and stratification Am J Kidney Dis 2002 39 2 Suppl 1 S1 266 11904577

[B7] Rule AD Larson TS Bergstralh EJ Slezak JM Jacobsen SJ Cosio FG Using serum creatinine to estimate glomerular filtration rate: accuracy in good health and in chronic kidney disease Ann Intern Med 2004 141 12 929 937 15611490 10.7326/0003-4819-141-12-200412210-00009

[B8] Stevens LA Coresh J Feldman HI Greene T Lash JP Nelson RG Rahman M Deysher AE Zhang YL Schmid CH Evaluation of the modification of diet in renal disease study equation in a large diverse population J Am Soc Nephrol 2007 18 10 2749 2757 10.1681/ASN.2007020199 17855641

[B9] Levey AS Coresh J Greene T Stevens LA Zhang YL Hendriksen S Kusek JW Van Lente F Using standardized serum creatinine values in the modification of diet in renal disease study equation for estimating glomerular filtration rate Ann Intern Med 2006 145 4 247 254 16908915 10.7326/0003-4819-145-4-200608150-00004

[B10] Levey AS Stevens LA Schmid CH Zhang YL Castro AF Feldman HI Kusek JW Eggers P Van Lente F Greene T A new equation to estimate glomerular filtration rate Ann Intern Med 2009 150 9 604 612 19414839 10.7326/0003-4819-150-9-200905050-00006 PMC2763564

[B11] Matsushita K Selvin E Bash LD Astor BC Coresh J Risk implications of the new CKD Epidemiology Collaboration (CKD-EPI) equation camopared with the MDRD Study equation for estimated GFR: the Atherosclerosis Risk in Communities (ARIC) study Am J Kidney Dis 2010 55 648 659 10.1053/j.ajkd.2009.12.016 20189275 PMC2858455

[B12] National Institute for Health and Clinical Excellence Guideline C673 Chronic kidney Disease 2008

[B13] Gaskin DJ Hoffman C Racial and ethnic differences in preventable hospitalizations across 10 states Med Care Res Rev 2000 57 Suppl 1 85 107 11092159 10.1177/1077558700057001S05

[B14] Choi AI Rodriguez RA Bacchetti P Bertenthal D Hernandez GT O'Hare AM White/black racial differences in risk of end-stage renal disease and death Am J Med 2009 122 7 672 678 10.1016/j.amjmed.2008.11.021 19559170 PMC2749005

[B15] Rostand SG Kirk KA Rutsky EA Pate BA Racial differences in the incidence of treatment for end-stage renal disease N Engl J Med 1982 306 21 1276 1279 10.1056/NEJM198205273062106 7040967

[B16] Levey AS Bosch JP Lewis JB Greene T Rogers N Roth D A more accurate method to estimate glomerular filtration rate from serum creatinine: a new prediction equation. Modification of Diet in Renal Disease Study Group Ann Intern Med 1999 130 6 461 470 10075613 10.7326/0003-4819-130-6-199903160-00002

[B17] Clase CM Garg AX Kiberd BA Prevalence of low glomerular filtration rate in nondiabetic Americans: Third National Health and Nutrition Examination Survey (NHANES III) J Am Soc Nephrol 2002 13 5 1338 1349 10.1097/01.ASN.0000013291.78621.26 11961022

[B18] Hunsicker LG Adler S Caggiula A England BK Greene T Kusek JW Rogers NL Teschan PE Predictors of the progression of renal disease in the Modification of Diet in Renal Disease Study Kidney Int 1997 51 6 1908 1919 10.1038/ki.1997.260 9186882

[B19] Hsu CY Lin F Vittinghoff E Shlipak MG Racial differences in the progression from chronic renal insufficiency to end-stage renal disease in the United States J Am Soc Nephrol 2003 14 11 2902 2907 10.1097/01.ASN.0000091586.46532.B4 14569100

[B20] Brancati FL Whittle JC Whelton PK Seidler AJ Klag MJ The excess incidence of diabetic end-stage renal disease among blacks. A population-based study of potential explanatory factors JAMA 1992 268 21 3079 3084 10.1001/jama.1992.03490210061036 1433738

[B21] Tarver-Carr ME Powe NR Eberhardt MS LaVeist TA Kington RS Coresh J Brancati FL Excess risk of chronic kidney disease among African-American versus white subjects in the United States: a population-based study of potential explanatory factors J Am Soc Nephrol 2002 13 9 2363 2370 10.1097/01.ASN.0000026493.18542.6A 12191981

[B22] Martins D Tareen N Norris KC The epidemiology of end-stage renal disease among African Americans Am J Med Sci 2002 323 2 65 71 10.1097/00000441-200202000-00002 11863081

[B23] Kopp JB Smith MW Nelson GW Johnson RC Freedman BI Bowden DW Oleksyk T McKenzie LM Kajiyama H Ahuja TS MYH9 is a major-effect risk gene for focal segmental glomerulosclerosis Nat Genet 2008 40 10 1175 1184 10.1038/ng.226 18794856 PMC2827354

[B24] Suthanthiran M Li B Song JO Ding R Sharma VK Schwartz JE August P Transforming growth factor-beta 1 hyperexpression in African-American hypertensives: A novel mediator of hypertension and/or target organ damage Proc Natl Acad Sci USA 2000 97 7 3479 3484 10.1073/pnas.050420897 10725360 PMC16265

[B25] Norris K Mehrotra R Nissenson AR Racial differences in mortality and ESRD Am J Kidney Dis 2008 52 2 205 208 10.1053/j.ajkd.2008.06.004 18640483 PMC2601720

[B26] Newsome BB McClellan WM Coffey CS Allison JJ Kiefe CI Warnock DG Survival advantage of black patients with kidney disease after acute myocardial infarction Clin J Am Soc Nephrol 2006 1 5 993 999 10.2215/CJN.01251005 17699318

[B27] Mehrotra R Kermah D Fried L Adler S Norris K Racial differences in mortality among those with CKD J Am Soc Nephrol 2008 19 7 1403 1410 10.1681/ASN.2007070747 18385428 PMC2440295

[B28] Popescu I Vaughan-Sarrazin MS Rosenthal GE Differences in mortality and use of revascularization in black and white patients with acute MI admitted to hospitals with and without revascularization services JAMA 2007 297 22 2489 2495 10.1001/jama.297.22.2489 17565083

[B29] Gao SW Oliver DK Das N Hurst FP Lentine KL Agodoa LY Sawyers ES Abbott KC Assessment of racial disparities in chronic kidney disease stage 3 and 4 care in the department of defense health system Clin J Am Soc Nephrol 2008 3 2 442 449 10.2215/CJN.03940907 18199843 PMC2390939

[B30] Delanaye P Cavalier E Mariat C Maillard N Krzesinski JM MDRD or CKD-EPI study equations for estimating prevalence of stage 3 CKD in epidemiological studies: which difference? Is this difference relevant? BMC Nephrol 2010 11 8 10.1186/1471-2369-11-8 20515483 PMC2891733

