# Supplementary material for: The MDRD equation underestimates the prevalence of CKD among blacks and overestimates the prevalence of CKD among whites compared to the CKD-EPI equation: a retrospective cohort study
Source: BMC Nephrol. 2012 Jan 20;13:4. doi: 10.1186/1471-2369-13-4 (PMC3398292; doi:10.1186/1471-2369-13-4)
Supplement: Additional file 1 — Table 4: Reclassification of CKD-EPI group by MDRD equation. Table 4 shows why there were significant differences in classification of stages of CKD using the 2 formulas. The patients who were initially classified in different eGFR categories by CKD-EPI method were reclassified again by applying MDRD equation. [file 1471-2369-13-4-S1.DOCX]

**Table 4: Reclassification of CKD-EPI group by MDRD equation**

**eGFR by MDRD**

| **eGFR by**  **CKD-EPI** | | | **Overall** | | **Black** | | **White** | |
| --- | --- | --- | --- | --- | --- | --- | --- | --- |
|  | | | % of patients classified in the next lower eGFR group by MDRD equation | % of patients classified in the next higher eGFR group by MDRD equation | % of patients classified in the next lower eGFR group by MDRD equation | % of patients classified in the next higher eGFR group by MDRD equation | % of patients classified in the next lower eGFR group by MDRD equation | % of patients classified in the next higher eGFR group by MDRD equation |
| No-CKD | | 90+ | ↓ 44.4% | 0% | ↓ 22% | 0% | ↓ 48.6% | 0% |
|  | | 60-89 | ↓ 5.7% | ↑2.3% | ↓0.9% | ↑ 0.9% | **↓ 6.0%** | ↑ 2.4% |
| CKD | 3a | 45-59 | ↓ 1.5% | ↑1.5% | ↓0.8% | **↑ 17.7%** | ↓ 1.6 | ↑ 0.6% |
|  | 3b | 30-44 | ↓ 0.2% | ↑ 14.8% | 0% | ↑ 22.8% | ↓ 0.3% | ↑ 14.4% |
|  | 4 | 15-29 | ↓ 0.56% | ↑ 17.3% | 0% | ↑1 9.6% | 0% | ↑17.2% |
|  | 5 | <15 | 0% | ↑9.85 | 0% | ↑6.7% | 0% | ↑ 10.7% |

Change in the classification of CKD stages by MDRD compared to CKD-EPI using 2 serum creatinine measurements. The percentage change in number of patients who are classified in the next lower eGFR group by MDRD equation is shown in the first column; the percentage of patients who are classified in the next higher eGFR group by MDRD equation is shown in the second column. For example, in black patients classified as eGFR 45-59 by CKD-EPI, 0.8% of these patients were classified as eGFR 30-44 (the next lower eGFR group) and 17.7% as eGFR 60-89 (the next higher eGFR group) by MDRD equation
